# Epiphytic microbiota source stimulates the fermentation profile and bacterial community of alfalfa-corn mixed silage

**DOI:** 10.3389/fmicb.2023.1247254

**Published:** 2024-04-02

**Authors:** Xiaolong Tang, Chaosheng Liao, Xiaokang Huang, Cheng Chen, Duhan Xu, Chao Chen

**Affiliations:** ^1^College of Animal Science, Guizhou University, Guiyang, China; ^2^Key Laboratory of Animal Genetics, Breeding and Reproduction in the Plateau Mountainous Region, Ministry of Education, Guizhou University, Guiyang, Guizhou, China

**Keywords:** epiphytic microbiota source, alfalfa, corn, fermentation profile, microbial diversity

## Abstract

The epiphytic microbiota source on plants plays a crucial role in the production of high-quality silage. To gain a better understanding of its contribution, the microbiota of alfalfa (M1C0), corn (M0C1) and the resulting mixture (M1C1) was applied in alfalfa-corn mixed silage production system. M1C0 decreased ammonia-N levels in terms of total nitrogen (57.59–118.23 g/kg TN) and pH (3.59–4.40) values (*p* < 0.01), which increased lactic acid (33.73–61.89 g/kg DM) content (*p* < 0.01). Consequently, this resulted in higher residual water-soluble carbohydrate (29.13–41.76 g/kg DM) and crude protein (152.54–167.91 g/kg DM) contents, as well as lower NDF (427.27 g/kg DM) and ADF (269.53 g/kg DM) contents in the silage compared to M1C1- and M0C1-treated samples. Moreover, M1C0 silage showed significantly higher bacterial alpha diversity indices (*p* < 0.05), including the number of observed species and Chao1 and Shannon diversity indices, at the later stages of ensiling. *Lactobacillus, Kosakonia* and *Enterobacter* were the dominant bacterial species in silages, with a relative abundance of >80%. However, the abundance of *Lactobacillus amylovorus* in M0C1- and M1C1-treated silage increased (*p* < 0.01) in the late stages of ensiling. These findings confirmed that the epiphytic microbiota source exerts competitive effects during anaerobic storage of alfalfa-corn mixed silage.

## 1 Introduction

Ensiling is a well-established and low-cost method for preserving fresh forage with high biological potential (Pakarinen et al., 2011). In the natural ensiling process, the epiphytic bacteria converting water soluble carbohydrates (WSC) in forage under anaerobic conditions, thereby producing organic acids (mainly lactic acid), which creates an acid environment to inhibits undesirable microorganisms (Dong et al., 2022), allowing the nutritional properties of forage to be preserved for a long time, this acidification inhibits the growth of spoilage microorganisms (Xu et al., 2017), thus allowing for long-term preservation of forage nutritional characteristics. Silage has become increasingly important worldwide due to its resilience against climatic variations. More recently, the role of the epiphytic microbiota source found on forage surface in silage fermentation has emerged as an important research hotspot.

The epiphytic microbiota source is a critical factor affecting silage quality. Previous studies have shown that inoculating epiphytic lactic acid bacteria (LAB) isolated from forage is more effective in promoting ensiling than using commercial bacterial fermentation starters (Yahaya et al., 2004). Wang et al. (2020) reported changes in the microbial community and fermentation quality of oat forage silage when using epiphytic microbiota source from maize and sorghum. Guo et al. (2015) reported that the napier grass silage showed lactic acid-type fermentation rather than acetic acid-type as usual. It is important to explore the dynamic changes occurring in the epiphytic microbiota source during ensiling (Xu et al., 2017). The composition of epiphytic microbiota source on fresh forage surface is highly heterogenous (Dong et al., 2022), being primarily composed of LAB, yeasts, and molds, and it varies considerably among different types of forage. The contribution of the epiphytic microbiota source to silage fermentation quality also varies. High-temperature-sterilized forage affect lactic acid, acetic acid, ammonia nitrogen and 2,3-butanediol content, while microbial origin only affect acetic acid content (Mogodiniyai Kasmaei et al., 2017). In this context, mixed silage has been proposed as an alternative to preserve nutrients and improve silage quality compared to conventional silage (Larsen et al., 2017), as the interactions among fermenting microorganisms are more complex.

Ensiling involves a variety of microorganisms and driven by the epiphytic microbiota source initially found on harvested grass. LAB is often associated with high-quality silage fermentation, whereas other microorganisms such as intestinal bacteria, clostridia, yeasts, and molds are undesirable. The majority of the bacteria involved in lactic acid fermentation of silage belong to the genera *Lactobacillus*, *Pedicoccus*, *Weissella* and *Leuconostoc* (Ni et al., 2018). Pahlow et al. (2003) reported that yeast, *Bacillus*, and *Panicus* compete with LAB for the utilization of water-soluble carbohydrates (WSC) in the fermentation substrate, leading to LAB growth inhibition. As silage fermentation progresses, the total relative abundance of *Levilactobacillus*, *Lactococcus* and *Weissella* decreases, whereas that of *Bacillus* and *Panicus* increases (Guan et al., 2018). The increase in the relative abundance of *Bacillus* indicates that silage becomes more susceptible to decay when exposed to air (Ávila and Carvalho, 2020). It is known that yeasts compete with LAB for sugars and lactic acid as energy sources during ensiling, which results in higher dry matter loss in silage (Carvalho et al., 2014). Furthermore, understanding the dynamics occurring in the epiphytic microbiota source during mixed silage fermentation is particularly challenging. Certain techniques have been used to obtain sterile substrates for silage production, among which are included γ-ray radiation (Heron et al., 1986), autoclaving (Graham et al., 1985) and heating (121°C, 20 min; Mogodiniyai Kasmaei et al., 2014). Heating and autoclaving can damage enzyme activity and the physical structure of raw materials, thus resulting in poor-quality silage, while γ-ray irradiation would not destroy the enzyme activity and the physical structure of raw materials (Ding et al., 2013). Wang et al. (2020) reported that γ-ray irradiation is the least likely method to cause change in the chemical composition of forage grass compared to heat or chemical sterilization. γ-ray irradiation to successfully isolate the contribution of epiphytic microbiota and chemical properties in alfalfa silage quality, avoiding changes in chemical composition during sterilization (Yang et al., 2019). Nevertheless, the current knowledge on the dynamics within the epiphytic microbiota from different forage sources during mixed silage fermentation is still scant. As these re-search technologies developed, we used γ-ray irradiation to irradiate alfalfa or corn to elucidate the adaptation process of epiphytic microbiota in alfalfa-corn mixed silage.

Therefore, the present study aimed to analyze fermentation quality and microbial succession patterns in gamma-irradiated alfalfa-corn mixed silage, and evaluate dynamic changes in the epiphytic microbiota source. Based on the findings discussed herein, it can be suggested that epiphytic microbiota source. From single-source or mixed-source forage has a positive effect on fermentation quality in mixed silage.

## 2 Materials and methods

### 2.1 Silage production

Alfalfa (*Medicago sativa L*, Gannong No. 9, peak flowering stage) and corn (*Zea mays L,* JinFuYu66 YNP64YNW02,wax ripening stage) were manually harvested in 10-cm above the soil in Jianggu Town, Zhenyuan City, Qiandongnan Miao and Dong Autonomous Prefecture, Guizhou Province China (N27.17°, E108.54°) in August 5, 2022, and immediately chopped into approximately 2 ~ 3 cm sections using a chopper. Approximately 500 g of alfalfa or corn was packed into polyethylene plastic bags (35 cm × 50 cm) and sealed in a vacuum sealer. Half of the bags containing alfalfa or corn were subjected to irradiation using a ^60^Co source at an absorbed dose of 25 kGy for 2 h to eliminate epiphytic microorganisms (Dong et al., 2019).

All bags were divided into the following treatments: (1) M1C1: non-irradiated alfalfa (50%) and non-irradiated maize (50%); (2) M1C0: non-irradiated alfalfa (50%) and irradiated corn (50%); (3) M0C1: irradiated alfalfa (50%) and non-irradiated corn (50%). The preparation of the aforementioned treatments was conducted under sterile conditions. A total of 54 samples were prepared (3 treatments × 6 storage periods × 3 replicates), and three bags from each treatment were opened at room temperature (>25°C) after 1, 3, 5, 7, 15, and 30 days of ensiling. Samples were collected for the analysis of chemical composition, fermentation characteristics, microbial populations, and bacterial community composition.

### 2.2 Determination of chemical composition of prepared mixed silages

Approximately 150 g of the sample obtained from each sample bag was weighed and dried at 65°C until a constant weight was achieved over a period of 72 h to determine dry matter (DM) content. The samples were then ground using a pulverizer and then passed through a 0.20-mm sieve prior to chemical composition analysis. Crude protein (CP) was determined according to the method described by AOAC (1990) using an automatic Kjeldahl nitrogen analyzer. The contents of neutral detergent fibers (NDF) and acid detergent fibers (ADF) were determined using the washing method as proposed by Van Soest et al. (1991) and referred to the GB/T 20806-2006 analytical standard. The content of water soluble carbohydrates (WSC) was determined using anthrone-sulfuric acid colorimetry. The buffering capacity was measured by electro-metric titration using a Model PHS-3E pH meter (Shanghai Precision & Scientific Instrument Co., Ltd.; Playne and McDonald, 1966).

We determine pH or other variables by shaking 20 g of the sample obtained from each sample bag was mixed with 180 mL of sterile distilled water for 30 min at 150 rpm on a shaker at 37°C and mixing the sample with sterile distilled water for 1 min using a juicer, then filtered through four layers of gauze. The obtained filtrate was centrifuged at 4,500 ×*g* for 15 min at 4°C, and the pH of the filtrate was measured using a pH meter (Model PHS-3E pH meter, Shanghai Precision & Scientific Instrument Co., Ltd.). Furthermore, the ammonia nitrogen (ammonia-N) content was determined using the phenol-sodium hypochlorite colorimetric method. Lactic acid (LA), acetic acid (AA), propionic acid (PA), and butyric acid (BA) were analyzed in approximately 5 mL of the supernatant by high performance liquid chromatography (HPLC; Li et al., 2018).

### 2.3 Microbial enumeration in prepared mixed silages

LAB, yeasts, molds and coliform bacteria were enumeration by plate culture method (Cai et al., 1999). LAB were enumerated on MRS agar plates (BEIJING AOBOXING BIO-TECH Co., Ltd., Beijing, China) incubated anaerobically at 37°C for 48 ~ 72 h. Yeasts and molds were enumerated on Bengal red agar plates (BEIJING AOBOXING BIO-TECH Co., Ltd., Beijing, China) after 72 h of incubation at 28°C. Coliform bacteria were enumerated on Eosin Methylene Blue Agar Plate (BEIJING AOBOXING BIO-TECH Co., Ltd., Beijing, China) after 18 ~ 24 h of incubation at 37°C.

### 2.4 Evaluation of bacterial community composition by full-length 16S rRNA gene sequencing

Total genomic DNA of each sample was extracted using the CTAB method. The purified DNA samples were diluted to a concentration of 1 ng/μL using sterile water. Specific primers with barcodes 27F (AGAGTTTGATCCTGGCTCAG) and 1492R (GNTACCTTGTTACGACTT) were used for conducting full-length 16S ribosomal RNA (rRNA) gene sequencing. PCR products with the same concentration were mixed, and the mixture was subjected to 2% agarose gel electrophoresis. The target bands were recovered using a gel recovery kit (Qiagen). The SMRT Bell library was constructed by ligating sequencing linkers to both ends of the amplified DNA fragment using a DNA binder, and purification selection was performed using AMpure PB magnetic beads. The constructed library was quantified using a Qubit fluorometer and then sequenced on the PacBio platform. Functional prediction, principal coordinate analysis (PCoA) and alpha diversity of the microbial community of silages were analyzed using the NovoMagic platform (Novogene Bio Technology Co., Ltd., Beijing, China). Raw sequencing data of bacterial community in silages prepared in this study have been submitted to the GenBank NCBI database under the accession number PRJNA973813.

### 2.5 Statistical analysis

Data were initially processed in Excel 2010 and then analyzed using SPSS software version 26.0 (IBM Corp., Armonk. NY, United States). Duncan’s test was performed to analyze changes in chemical composition, microbial population, and bacterial community indices during forage fermentation for silage production, and multiple comparisons were performed. Spearman correlation analysis was conducted to explore the relationship between fermentation parameters and bacterial community composition. Statistical significance was considered when *p* values were < 0.05.

## 3 Results and discussion

### 3.1 Chemical composition of raw materials of single-source silage

Table 1 depicts the chemical composition and microbial composition of fresh and sterilized raw materials before ensiling. Fresh alfalfa was harvested at the peak flowering stage with DM content of 256.96 g/kg, which was below the recommended DM range of 300–400 g/kg fresh matter (FM; Yuan et al., 2017). WSC content (>50.00 g/kg DM) is a crucial factors to affect the speed and extent of silage fermentation (Ni et al., 2018). WSC content in fresh alfalfa was 58.71 g/kg FM, which was sufficient to ensure the smooth progress of silage fermentation. In addition, NDF, ADF and buffering capacity in fresh alfalfa were 438.60 g/kg DM, 323.40 g/kg DM and 478.00 mEq/kg DM, respectively. Corn was harvested at the wax ripening stage exhibited DM, WSC, NDF, ADF and buffering capacity of 257.54 g/kg FM, 125.93 g/kg DM, 528.20 g/kg DM, 338.20 g/kg DM and 231.00 mEq/kg DM, respectively. No significant differences were observed in DM, WSC, NDF and ADF contents in fresh alfalfa-corn or γ-ray irradiation mixed silage and γ-ray irradiation alfalfa-corn mixed silage, thus indicating that γ-ray irradiation in forage silage did not affect the chemical composition with alfalfa and corn, which is in accordance with the study of Yang et al. (2019). A study by Oliveira et al. (2017) reported that a LAB load >5.0 log_10_ cfu/g FM led to improved silage quality. The initial counts of yeasts and coliform bacteria in fresh alfalfa were 5.47 log_10_ cfu/g FM and 6.09 log_10_ cfu/g FM, respectively, and whereas in fresh corn they were 6.31 log_10_ cfu/g FM and 5.83 log_10_ cfu/g FM, respectively. After 25 kGy of γ-ray irradiation for 2 h, no LAB, yeasts, or coliform bacteria were detected in alfalfa-corn mixed silage, thus indicating the efficacy of γ-ray irradiation for forage sterilization.

**Table 1:** Chemical composition and microbial population composition of fresh and sterilized alfalfa and corn before ensiling.

Parameters	No γ-ray irradiation		γ-Ray irradiated		SEM	*p*-value
	Alfalfa	Corn	Alfalfa	Corn		
DM, g/kg FM	256.96	257.54	256.73	257.33	0.004	<0.001
WSC, g/kg DM	58.71B	125.93A	58.43B	125.51A	0.003	<0.001
CP, g/kg DM	190.53A	104.73B	188.2A	103.86B	0.004	<0.001
NDF, g/kg DM	438.63B	528.33A	438.20B	528.00A	0.028	<0.001
ADF, g/kg DM	323.43B	338.30A	323.63B	338.50A	0.037	<0.001
pH	6.18A	4.87B	6.16A	4.81C	0.005	<0.001
Buffering capacity, mEq/kg DM	478.33A	231.67B	475.67A	226.33C	0.264	<0.001
LAB, Log_10_ cfu/g FM	6.35A	6.05B	ND	ND	<0.001	<0.001
Yeasts, Log_10_ cfu/g FM	5.47B	6.31A	ND	ND	<0.001	<0.001
Coliform bacteria, Log_10_ cfu/g FM	6.09A	5.83B	ND	ND	<0.001	<0.001

DM, dry matter; FM, fresh matter; WSC, water soluble carbohydrates; CP, crude protein; NDF, neutral detergent fiber; ADF, acid detergent fiber; LAB, lactic acid bacteria; ND, not detected; SEM, standard error of mean. ^A–C^Indicate differences between values within the same row at *p* < 0.05.

### 3.2 Chemical composition of alfalfa-corn mixed silage

Table 2 depicts an overview of the chemical composition of alfalfa-corn mixed silage during ensiling. Alfalfa-corn mixed silage during silage time, treatment and their interactions significantly affect WSC, CP, pH, ammonia-N, lactic acid, acetic acid, propionic acid (*p* < 0.05), but had no effect on DM, NDF and ADF (*p* > 0.05). Changes in DM content during ensiling were considered indicators for evaluating high-quality silage, and an indication the amount of water required for microbial growth. The nutrient content in raw material for silage, especially the dry matter content, is a key factor to affect the quality of silage fermentation (Xu D. M. et al., 2018). Throughout the storage period, the DM content in M1C1-, M1C0- and M0C1-treated samples showed an upward trend, with the highest DM content in observed in M1C0-treated samples. Moreover, a decrease in WSC content in all treatments (*p* < 0.001). However, both NDF and ADF contents showed initially an upward trend and then decreased. After 30 days of ensiling, the ADF content in M1C1- and M0C1-treated samples was significantly higher compared to M1C0-treated samples (*p* < 0.001), likely due to the consumption of digestible nutrients by microorganisms.

**Table 2:** Fermentation characteristics of alfalfa-corn mixed silage during ensiling.

Parameters	Treatment (T)	Ensiling time (E)						SEM	*p*-value		
		Day 1	Day 3	Day 5	Day 7	Day 15	Day 30		T	E	T × E
DM, g/kg FM	M1C1	228.99	229.11	235.51	234.76	238.59	241.13	0.348	<0.001	<0.001	0.507
	M1C0	234.89	236.07	236.26	237.83	242.26	247.68				
	M0C1	228.53	230.42	232.68	236.43	239.12	241.5				
WSC, g/kg DM	M1C1	33.94Ca	33.57Ca	33.20Ca	30.24Cb	28.15c	28.11c	0.207	<0.001	<0.001	<0.001
	M1C0	41.76Ba	40.28Ba	35.79Bb	32.92Bc	32.51c	29.13d				
	M0C1	48.52Aa	44.73Aab	42.55Ab	38.29Ac	33.11d	29.68d				
CP, g/kg DM	M1C1	156.63Ca	154.74a	154.23Aa	151.44Bb	149.66ABb	149.77Bb	0.164	<0.001	<0.001	<0.001
	M1C0	167.91Aa	156.47b	153.67ABc	153.16Ac	151.27Ad	152.54Acd				
	M0C1	161.12Ba	154.08b	153.21Bb	152.23ABb	147.49 Bd	149.90Bc				
NDF, g/kg DM	M1C1	478.4	502.2	479.13	476.73	463.73	462.47	3.233	0.399	0.001	0.418
	M1C0	479.8	499.67	497.2	490.87	431.07	427.27				
	M0C1	476.09	500.22	489.76	484.69	456.24	452.82				
ADF, g/kg DM	M1C1	284.87	296.27	288	287.8	286.8	278.8	2.482	0.437	0.001	0.311
	M1C0	274.4	313.47	310.6	297.07	290.33	269.53				
	M0C1	291.93	305.47	303.73	295.27	257.87	255				
pH	M1C1	4.69Aa	4.51Ab	4.19Ac	4.19Ac	3.99Ad	3.93Ad	0.004	<0.001	<0.001	<0.001
	M1C0	4.40Ca	4.05Cb	3.91Bc	3.79 Bd	3.61Ce	3.59Ce				
	M0C1	4.58Ba	4.35Bb	4.23Ac	4.17Ad	3.79Be	3.78Be				
Ammonia-N, g/kg TN	M1C1	61.59Ad	99.10Ac	100.55Ac	133.75Ab	142.86Aa	134.27Ab	0.412	<0.001	<0.001	<0.001
	M1C0	57.59 Bd	68.76Bc	88.94Bb	99.01Cb	120.32Ba	118.23Ca				
	M0C1	61.04Ae	92.62Ad	97.11Ad	117.59Bc	144.02Aa	127.59Bb				
Lactic acid, g/kg DM	M1C1	20.12Bc	21.72Bbc	23.07Bbc	24.59Bbc	27.66Cab	33.61Ba	0.485	<0.001	<0.001	0.006
	M1C0	33.73Ad	34.64Ad	36.30Acd	40.80Ac	50.18Ab	61.89Aa				
	M0C1	21.93Bc	24.32Bc	28.53ABbc	29.39Bbc	34.23Bab	37.29Ba				
Acetic acid, g/kg DM	M1C1	6.84d	8.36Acd	8.95Abcd	9.32bc	11.20Bab	12.87Aa	0.158	<0.001	<0.001	0.035
	M1C0	5.99b	6.09Bb	6.29Bb	6.87b	8.63Ca	9.89Ba				
	M0C1	7.87d	9.03Acd	9.44Abcd	10.76bc	14.96Aa	11.54ABb				
Propionic acid, g/kg DM	M1C1	0.00d	8.78Ac	8.97Ac	10.03Ac	13.47Bb	22.27Aa	0.16	<0.001	<0.001	<0.001
	M1C0	0.00b	0.00Bb	0.00Bb	0.00Bb	8.13Ca	0.00Bb				
	M0C1	0.00d	0.00 Bd	9.61Ac	13.12Ab	19.19Aa	20.72Aa				

DM, dry matter; FM, fresh matter; WSC, water soluble carbohydrates; CP, crude protein; NDF, neutral detergent fiber; ADF, acid detergent fiber; M1C1, non-irradiated alfalfa (50%) and non-irradiated corn (50%); M1C0, non-irradiated alfalfa (50%) and irradiated corn (50%); M0C1, irradiated alfalfa (50%) and non-irradiated corn (50%); SEM, standard error of mean; ^A–C^Indicate differences between values within the same column at *p* < 0.05; ^a–d^Indicate differences between values within the same row at *p* < 0.05.

The pH of silage is an important indicator reflecting the utilization rate of WSC by microorganisms. McDonald et al. (1991) reported that the pH of high-quality silage should be below 4.2. Throughout the storage period, the pH of M1C1-, M1C0- and M0C1-treated silage showed a downward trend (*p* < 0.001). In particular, the pH of M1C0- and M0C1-treated silage was significantly lower than that of M1C1-treated silage (*p* < 0.001), which was consistent with the findings of McDonald et al. (1991). Moreover, the pH decrease of silage primarily occurred during the initial 15 days of ensiling, and did not significantly change as the storage period progressed, which was consistent with the findings of Ni et al. (2017a).

During the silage process, crude protein degradation is unavoidable, and some true protein is converted to non-protein-N (especially ammonia-N) under the action of plant enzymes and microorganisms, thereby reducing the utilization of feed protein by ruminants and increasing nitrogen emissions in animal production (He et al., 2020). Crude protein was significantly reduced during the fermentation process (*p* < 0.001). Ammonia-N is an important indicator reflecting protein hydrolysis in silage. Meanwhile, ammonia-N is produced by the activity of fermentative coliform bacteria or plant proteases (Ogunade et al., 2018). In the present study, the proportion of ammonia-N to total-N increased linearly in the initial 15 days of ensiling (*p* < 0.001) and then stabilized in M1C0-treated silages. However, the proportion of ammonia-N to total-N in each treatment decreased after 30 days of ensiling. M1C0-treated improves microbial fermentation and protein preservation in silage and reduces ammonia nitrogen production. In addition, M1C0-treated silage exhibited the lowest the ammonia-N content during ensiling, which indicated the lowest rate of protein degradation.

The decline in silage pH during fermentation is mainly caused by the production of organic acid. As shown in Table 1, lactic acid and acetic acid were the dominant organic acids during ensiling. Within 30 days of ensiling, the lactic acid content significantly increased in sterilized silage (*p* < 0.05). Compared to M1C0-treated silage, the lactic acid content in M1C1- and M0C1-treated silage was significantly lower (*p* < 0.05). However, the lactic acid content in M0C1-treated silage was lower than that in M1C0-treated silage (*p* < 0.05) but higher than that in M1C1-treated silage. These observations revealed that lactic acid production was primarily attributed to the epiphytic microbiota associated with alfalfa. Compared to M1C0- and M0C1-treated silage, the lactic acid content was lower in M1C1-treated silage, thus indicating the antagonistic effects established during fermentation by the epiphytic microbiota from the two forage grasses. Compared to M0C1-treated silage, the epiphytic microbiota from alfalfa in M1C0-treated silage had a promoting effect on lactic acid fermentation, whereas the epiphytic microbiota from corn had an inhibitory effect.

Furthermore, the acetic acid content gradually increased during ensiling (*p* < 0.001). According to Pahlow et al. (2003), under low DM content and high buffering capacity, lactic acid and propionic acid produced are converted into acetic acid. The propionic acid content (*p* < 0.001) gradually increased after 30 days of ensiling, except for M1C0-treated silage. The increase in acetic acid and propionic acid contents with prolonged ensiling time is consistent with previous studies (Jahanzad et al., 2016).

Finally, butyric acid was not detected in silages, which could be attributed to the production of large amounts of lactic acid and acetic acid during the initial 30 days of ensiling, thus preventing coliform bacteria from fermenting sugars into butyric acid or converting lactic acid into butyric acid (Kung et al., 2018).

### 3.3 Microbial population composition of alfalfa-corn mixed silage

Silage fermentation is initiated and regulated by microorganisms. As shown in Table 3, the microbial composition of the evaluated silages was examined. Ensiling led to an increase LAB counts in silages (*p* < 0.001). In particularly, LAB counts in M1C1- and M1C0-treated silage decreased during the initial 7 days of ensiling and then increased after 15 days of ensiling. Kung et al. (2018) reported that yeasts and coliform bacteria competed with LAB for WSC as fermentation substrates during ensiling. The counts of yeasts, coliform bacteria, and molds in all samples exceeded the detection level (<2.0 log_10_ cfu/g FM). After 30 days of ensiling, the population of yeasts, coliform bacteria, and molds in M1C1- and M0C1-treated silage was higher than that in M1C0-treated silage (*p* < 0.001). This suggests that the M1C0 treatment effectively inhibited the growth of yeasts, coliform bacteria, and molds, thus creating a favorable condition for the proliferation of LAB.

**Table 3:** Effects of different treatments on lactic acid bacteria, fungi, coliform bacteria count counts in alfalfa-corn mixed silage during ensiling.

Parameters	Treatment (T)	Ensiling time (E)						SEM	*p*-value		
		Day 1	Day 3	Day 5	Day 7	Day 15	Day 30		T	E	T × E
Lactic acid bacteria, Log_10_cfu/g FM	M1C1	6.29b	6.10Cc	5.89Ad	5.73d	6.46Aab	6.56a	0.02	0.004	<0.001	<0.001
	M1C0	6.22b	6.16Bb	5.75Bc	5.71c	6.32Ab	6.78a				
	M0C1	6.11ab	6.24Aa	5.83ABbc	5.87bc	5.58Bc	6.43a				
Yeasts and modes, Log_10_cfu/g FM	M1C1	5.89a	4.54Bb	4.50ABb	4.17Bc	4.04c	4.24bc	0.041	<0.001	<0.001	0.001
	M1C0	5.71a	4.39Bbc	4.33Bbc	4.82Ab	4.58bc	4.15c				
	M0C1	5.79a	5.57Aab	5.04Abc	4.40ABc	4.44c	5.14abc				
Coliform bacteria, Log_10_cfu/g FM	M1C1	5.80a	5.42Ab	4.99Ab	4.91Abc	4.61Abc	4.51Ac	0.025	<0.001	<0.001	<0.001
	M1C0	5.88a	5.11Bb	5.01Ab	4.47Bc	4.11 Bd	3.94 Bd				
	M0C1	6.11a	4.35Cc	4.08Bc	4.35Bc	4.65Ab	4.33ABc				

M1C1, non-irradiated alfalfa (50%) and non-irradiated corn (50%); M1C0, non-irradiated alfalfa (50%) and irradiated corn (50%); M0C1, irradiated alfalfa (50%) and non-irradiated corn (50%); SEM, standard error of mean; ^A–C^Indicate differences between values within the same column at *p* < 0.05; ^a–d^Indicate differences between values within the same row at *p* < 0.05.

### 3.4 Alpha diversity in the bacterial community of alfalfa-corn mixed silage

Table 4 depicts the results of alpha diversity analysis of the bacterial community in silage samples. Less than 99% of Good’s coverage index indicated that the depth of high-throughput se-quencing was satisfactory for the microbial compositions in silage samples and was enough for credible analysis. It is well known that beneficial bacteria contribute rapidly dominate the microbiota to improve silage quality in the middle and late stages (Ren et al., 2019). The Shannon diversity index varied significantly during ensiling, ranged from 1.14 to 4.34 (*p* < 0.001). Decreased in bacterial diversity and richness in M1C1-treated silage during the early stage of ensiling, which is the same finding by Xu Z. S. et al. (2018). The highest Shannon index (3.64–4.21) was observed during the initial 15 days in M1C1-treated silage. Comparisons between untreated soybean silage and pre-silage have shown that pre-silage showed higher bacterial diversity (Ni et al., 2017b). This could be due to the relatively high pH value of M1C1-treated silage, which was not sufficiently low to inhibit microbial growth. The Chao1 index was used to measure species richness in the microbial community of silages, which was found increased and then decreased in M1C1-treated during ensiling (*p* < 0.05). Compared to M1C0-treated, the decrease in Chao1 index in M0C1 silage was greater (*p* < 0.05).

**Table 4:** Effects of different treatments on bacterial alpha-diversity indices in alfalfa-corn mixed silage during ensiling.

Indices	Fresh forage		Treatment (T)	Ensiling time (E)						SEM	*p*-value		
	Alfalfa	Corn		Day 1	Day 3	Day 5	Day 7	Day 15	Day 30		T	E	T × E
Observed species	307.00	184.00	M1C1	167.67Aabc	176.67Aab	181.33Aa	148.67ABbc	145.00Ac	101.00 Bd	3.299	<0.001	<0.001	0.043
			M1C0	141.67AB	126B	153AB	160.00A	162.33A	143.33A				
			M0C1	109.67Bab	105Bb	139Ba	131.33Bab	110.67Bab	44.33Cc				
Shannon	4.68	3.71	M1C1	4.21Aa	4.20Aa	4.12a	3.64a	4.01a	2.90Bb	0.05	0.001	<0.001	<0.001
			M1C0	3.66B	3.47B	3.91	3.92	3.81	4.15A				
			M0C1	3.76ABb	3.75Bb	4.34a	3.90b	3.35c	1.14Cd				
Chao1	322.19	205.48	M1C1	202.22ab	211.35Aa	215.1Aa	168.6ABbc	159.74Bc	111.82ABd	4.263	<0.001	<0.001	0.043
			M1C0	183.76	148.48B	180.34AB	194.78A	188.34A	167.40A				
			M0C1	126.86c	120.68Bc	165.15Ba	159.78Bab	139.30Cbc	51.50Cd				
Good’s coverage	0.996	0.997	M1C1	0.995	0.995	0.995	0.996	0.997	0.998	<0.001	<0.001	0.003	0.034
			M1C0	0.995	0.997	0.996	0.995	0.996	0.996				
			M0C1	0.997	0.998	0.997	0.996	0.996	0.999				

M1C1, non-irradiated alfalfa (50%) and non-irradiated corn (50%); M1C0, non-irradiated alfalfa (50%) and irradiated corn (50%); M0C1, irradiated alfalfa (50%) and non-irradiated corn (50%); SEM, standard error of mean. ^A–C^Indicate differences between values within the same column at *p* < 0.05; ^a–d^Indicate differences between values within the same row at *p* < 0.05.

**Figure 1:**
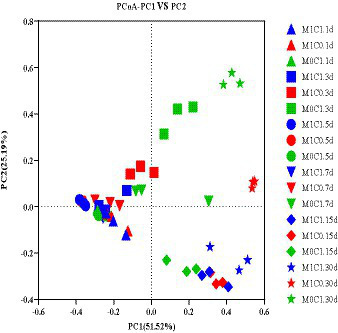
Principal coordinate analysis (PCoA) of the bacterial community structure of alfalfa-corn mixed silage. M1C1, non-irradiated alfalfa (50%) and non-irradiated maize (50%); M1C0, non-irradiated alfalfa (50%) and irradiated corn (50%); M0C1, irradiated alfalfa (50%) and non-irradiated corn (50%).

The difference of the bacterial community composition was markedly described in the principal co-ordinates analysis (PCoA) plot. As shown in Figure 1, PCoA analysis revealed that principal component (PC) 1 and principal component (PC) 2 explained 51.52 and 25.19% of the difference in bacterial community composition, respectively. M1C1-, M1C0-and M0C1-treated silages clustered separately, which showed that γ-ray irradiation altered the bacterial community structure of silage.

Yang et al. (2019) evaluated the addition of *Lactobacillus plantarum* to untreated and sterilized alfalfa silage, and showed that gamma irradiation did not affect the bacterial community composition. Ni et al. (2017b) reported that, with prolonged ensiling, non-LAB species of the epiphytic microbiota were inhibited or inactivated in pre-ensiled Italian ryegrass and crop corn. In the present study, the bacterial communities of M1C1- and M0C1-treated silages were similar, unlike M1C0-treated silage. Taken together, these results showed that γ-ray irradiation had significant impact on the composition of surface epiphytic microbiota in alfalfa-corn mixed silage.

### 3.5 Bacterial community composition of alfalfa-corn mixed silage

**Figure 2:**
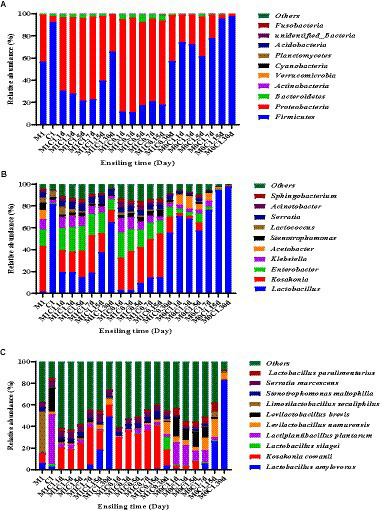
Effects of different treatments on the relative abundance of the first ten phyla **(A)**, genus **(B)**, and species **(C)** in alfalfa-corn mixed silage. M1C1, non-irradiated alfalfa (50%) and non-irradiated corn (50%); M1C0, non-irradiated alfalfa (50%) and irradiated corn (50%); M0C1, irradiated alfalfa (50%) and non-irradiated corn (50%).

Parvin et al. (2010) described several microbial communities and succession patterns in different silages, which contributed to a better understanding of the complex ensiling process by evaluating microbial community composition (Xu D. M. et al., 2018). Changes in bacterial community composition of alfalfa-corn mixed silage at the phylum level are shown in Figure 2A. At the phylum level, *Firmicutes* followed by *Proteobacteria* dominated in fresh alfalfa and corn, with a higher relative abundance of *Firmicutes* in corn.

At the genus level (Figure 2B), the relative abundance of *Lactobacillus* increased with prolonged ensiling time, whereas the relative abundance of *Enterobacter* and *Kosakonia* gradually decreased. Interestingly, the relative abundance of *Lactobacillus* in M1C0-treated increased linearly (*p* < 0.01). *Enterobacter* and *Kosakonia* were found in M1C1- and M1C0-treated silages. It may be that *Kosakonia* has a strong acid resistance, which remains to be verified. In the early stages of ensiling, certain acid-producing cocci conduct lactic acid fermentation. In contrast, in the late stages of ensiling, LAB with strong acid-resistant ability plays an important role in reducing the pH of silage (Cai et al., 1998).

**Figure 3:**
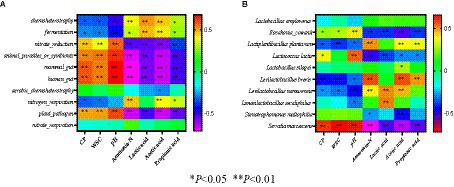
**(A)** Spearman correlation between microbiota dominant bacteria and silage fermentation parameters, **(B)** Spearman correlation between functional predictions and silage fermentation parameters.

At the species level (Figure 2C), *Lactobacillus plantarum* played a crucial role in promoting fermentation during the early stages of ensiling, produced lactic acid and decreased pH to inhibit the growth of undesirable microorganisms and prevents further decomposition of sugars and proteins in silage (Yan et al., 2019). The relative higher abundance of *Lactobacillus plantarum* in fresh corn can explain its abundance in M0C1 silage. In M0C1 silage, the relative abundance of *L. plantarum* gradually decreased from day 1 to day 30 of ensiling. *Lactobacillus brevis* is an obligatory heterofermentative LAB species which produces high acetic acid contents, which can improve silage aerobic stability and prevent deterioration caused by undesirable microorganisms (Guo et al., 2017). *Lactobacillus brevis* was found in all samples of M1C1-, M1C0- and M0C1-treated, which explained the high acetic acid contents. Compared to M0C1-treated, *Kosakonia cowanii* was observed in high relative abundance in M1C1- and M1C0-treated. As shown in Figure 3, pH value was negatively correlated with *Kosakonia cowanii* counts (*p* < 0.01), thus indicating that *Kosakonia cowanii* had strong acid resistance ability. However, further studies are required to elucidate whether *Kosakonia cowanii* has acid-producing properties.

### 3.6 Correlation analysis and function prediction analysis of silage quality parameters and bacterial community composition of alfalfa-corn mixed silage

**Figure 4:**
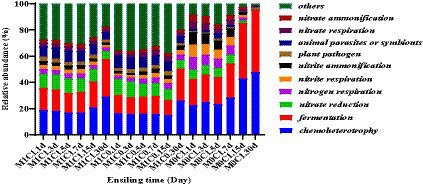
Function prediction of the bacterial community of mixed silage during silage fermentation.

The function prediction analysis of the bacterial community of silage is shown in Figure 4. The main functions of the bacterial community identified during ensiling of alfalfa-corn mixed silage were chemical heterotrophic, fermentation, and nitrate reduction. Chemical heterotrophic and fermentation increased gradually in the late stages of ensiling, with greater changes in M0C1-treated silages compared to M1C1- and M1C0-treated. This increase in chemical heterotrophy and fermentation may be attributed to the degradation of starch by *Lactobacillus amylovorus* during the late stages of ensiling, leading to the production of WSC. Zhang et al. (2018) reported that with the increase in chemoheterotrophy and fermentation, most bacteria utilize carbon and energy for oxidizing organic components for carbon fixation. Moreover, Li et al. (2021) reported that a correlation between the continuous production of ammonia-N and nitrogen respiration, nitrite ammonification and nitrite respiration. Higher rates of nitrogen respiration, nitrite ammonification and nitrite respiration were detected in all silage samples, which explained the continuous generation of ammonia-N.

Figure 3 depicts Spearman correlation coefficients between dominant bacteria /function prediction of microbiota and silage fermentation parameters. The WSC content was negatively correlated with *Lactobacillus plantarum* in silage samples (*p* < 0.01), indicating that *Lactobacillus plantarum* utilizes WSC as a fermentation substrate (Chen et al., 2020). This could explain the decrease in WSC. It is well known that at the late stages of ensiling, *Lactobacillus* plays an important role in increasing the lactic acid content and reducing pH value (Cai et al., 1998). The pH value was negatively (*p* < 0.01) correlated with the abundance of *Lactobacillus* species (including *Lactobacillus plantarum*, *Lactobacillus brevis*, *Lactobacillus namurensis* and *Lactobacillus secaliphilus*), but positively (*p* < 0.01) correlated with *Kosakonia cowanii* and *Serratia marcescens*. Ammonia-N production was positively correlated with the abundance of *Lactobacillus brevis*, *Lactobacillus plantarum* and *Lactobacillus namurensis*, indicating that chemoheterotrophy and fermentation continued slowly, thus allowing for the consumption of WSC and CP during ensiling, which are in agreement with the results reported by Li et al. (2022). Finally, the results revealed that the accumulation of lactic acid was positively correlated with the presence of *Lactobacillus namurensis* and *Lactobacillus secaliphilus* (*p* < 0.01).

## 4 Conclusion

This study highlights the antagonistic effects of the epiphytic microbiota source from alfalfa and corn during the fermentation process of mixed silage. The epiphytic microbiota source from alfalfa promoted lactic acid fermentation, while epiphytic microbiota source from corn inhibited acetic acid fermentation and had a positive impact on propionic acid fermentation. Therefore, understanding the role of the epiphytic microbiota source in mixed silage fermentation quality and the related dynamic changes occurring among microorganisms is crucial. The findings of the present work contribute to the knowledge base of mixed silage production.

## Data availability statement

Raw sequencing data of bacterial community in silages prepared in this study have been submitted to the GenBank NCBI database under the accession number PRJNA97381.

## Author contributions

XT and CL: data curation, formal analysis, visualization, writing – original draft, and writing – review and editing. XH, CheC, and DX: investigation. ChaC: conceptualization, methodology, validation, writing – review and editing, supervision, funding acquisition, project administration, and funding acquisition. All authors contributed to the article and approved the submitted version.
